# The association of cognitive task scores with energy intake measurement error from technology-assisted 24-h recalls

**DOI:** 10.1017/S000711452500042X

**Published:** 2025-03-28

**Authors:** Clare Whitton, Barbara A. Mullan, Satvinder S. Dhaliwal, Richard Norman, Carol J. Boushey, Clare E. Collins, Megan E. Rollo, Deborah A. Kerr

**Affiliations:** 1 Curtin School of Population Health, Curtin University, Kent Street, GPO Box U1987, Perth 6845, WA, Australia; 2 Curtin Medical Research Institute, Curtin University, Kent Street, GPO Box U1987, Perth 6845, Australia; 3 School of Medical and Health Sciences, Edith Cowan University, 270 Joondalup Drive, Joondalup, WA 6027, Australia; 4 Enable Institute, Curtin University, Kent Street, GPO Box U1987, Perth 6845, Australia; 5 Obstetrics & Gynaecology Academic Clinical Program, Duke-NUS Medical School, National University of Singapore, 8 College Rd, 169857, Singapore; 6 Health & Social Sciences, Singapore Institute of Technology, 1 Punggol Coast Road, 828608 Singapore; 7 Office of the Provost, Singapore University of Social Sciences, 463 Clementi Road, 599494 Singapore; 8 Epidemiology Program, University of Hawaii Cancer Center, Honolulu, HI, USA; 9 School of Health Sciences, College of Health, Medicine and Wellbeing, University of Newcastle, Newcastle, Australia; 10 Food and Nutrition Research Program, Hunter Medical Research Institute, New Lambton Heights, Newcastle, Australia

**Keywords:** Dietary assessment, Measurement error, Neurocognitive processes, Executive function

## Abstract

Measurement error undermines the accuracy of dietary intake data. The 24-h dietary recall (24HR) is the standard data collection method in nutrition surveillance. Several neurocognitive processes underpin the act of recall, and individuals differ in their performance of these processes. This study aimed to investigate whether variation in neurocognitive processes, measured using four cognitive tasks, was associated with variation in measurement error of 24HR. Participants (*n* 139) completed the Trail Making Test, the Wisconsin Card Sorting Test, the Visual Digit Span and the Vividness of Visual Imagery questionnaire. During a controlled feeding study, participants completed three technology-assisted 24HR: the Automated Self-Administered Dietary Assessment Tool, Intake24 and an Interviewer-Administered Image-Assisted 24HR (IA-24HR) 1 week apart. The percentage error between reported and true energy intakes was calculated. Using linear regression, the association between cognitive task scores and absolute percentage error in estimated energy intake was assessed. Longer time spent completing the Trail Making Test, an indicator of visual attention and executive functioning, was associated with greater error in energy intake estimation using ASA24 (B 0·13, 95 % CI 0·04, 0·21) and Intake24 (B 0·10, 95 % CI 0·02, 0·19). Regression models explained 13·6 % (ASA24) and 15·8 % (Intake24) of the variance in energy estimation error. No cognitive task scores were associated with error using IA-24HR. This study demonstrates that variation between individuals in neurocognitive processes explains some of the variation in 24HR error. Further investigation into the role of neurocognitive processes in 24HR and their role in the reliability of dietary intake data is warranted.

Recalling dietary intake is a central part of population nutrition surveillance conducted to inform public health nutrition policy and interventions. The 24-h dietary recall (24HR) method is a standard method in nutrition surveillance^(1–5)^, during which participants receive temporal cues and content cues to retrieve memories^(6)^ and are subsequently required to recall, describe and quantify all the foods and beverages they have consumed in the previous 24 h. Knowledge about human cognition has assisted the development of 24HR methods to optimise the accuracy of data collection, such as the multiple-pass method, in which there are multiple rounds of probing in relation to each eating occasion^(7)^. Although the 24HR method results in lower measurement error compared with other instruments, error remains an issue. A recent systematic review of dietary assessment validation studies indicated that 24HR underestimated energy intake by 8–30 %^(8)^. This may be related in part to the cognitive challenges involved in completing a 24HR. The act of recalling, describing and quantifying involves several cognitive processes, including perception, memory, conceptualisation of that memory, numeracy, recall and the formulation of a response^(9–11)^. Individuals differ in their performance of cognitive processes^(12)^, but to date, it is unknown whether this variation contributes to measurement error within the 24HR process.

Errors in dietary reporting can occur in the encoding and/or retrieval of memories and in the mapping of those memories into a response^(6)^. The encoding of memory is influenced by attention, perception or interpretation (i.e. whether the individual perceives the item to be what it actually was), organisation (how the memory is labelled) and retention^(10)^. Paying attention during encoding of a memory results in better subsequent recall^(13,14)^. For example, paying attention to food while eating has resulted in a more vivid memory of the meal later that day^(14)^. In contrast, divided attention during encoding of a memory has been associated with large reductions in subsequent recall of that memory^(15)^. Some evidence suggests that the strength of visual imagery predicts memory capacity^(16)^, while some studies have found no association between visual imagery and visual short-term memory^(17)^. Once memories are encoded, various processes are involved in the retrieval of those memories and the formulation of responses about the description and amount of food/beverage consumed^(10)^. For example, cognitive flexibility allows an individual to switch cognitive strategies and consider two or more aspects of an object, idea or complex situation simultaneously^(18)^. Another aspect of cognitive flexibility is the ability to change perspectives spatially and imagine what something would look like from another angle or direction^(19)^. Despite the clear role of these cognitive processes in a 24HR, none has been measured quantitively in studies investigating dietary intake measurement error.

Previous research on factors affecting dietary intake measurement error has focussed on demographic and psychosocial attributes. For example, being a woman, having a higher BMI, smoking behaviour and lower socio-economic status have been associated with a greater likelihood of measurement error^(8,20–22)^. Of the many psychosocial attributes studied such as dietary restraint^(23)^ and fear of negative evaluation^(24)^, social desirability bias appears to be the most consistently identified bias within self-report^(11)^. In terms of cognitive factors and their association with dietary intake measurement error, two studies separately assessed the impact on reporting error of perception at the time of eating *v*. the conceptualisation of memory and found that both factors contributed to reporting error^(25,26)^. In a study of Welsh children aged 9–11, better episodic memory was associated with omitting fewer items from a self-administered questionnaire on intake of foods at breakfast^(27)^, but no associations were found with working memory or attention. In another study among children, school test scores were used as a proxy for cognitive ability, and higher test scores were associated with a decrease in dietary reporting error^(28)^. Overall, research on cognitive factors in relation to dietary intake measurement error, particularly among adults, is sparse.

Individual differences in visual attention, short-term and working memory, conceptualisation and response formulation may contribute to between-person variation in 24HR measurement error. Therefore, the aim of the present study was to investigate whether variation in neurocognitive processes measured using cognitive tasks could predict variation in the error in self-reported 24HR.

## Methods

### Sample and recruitment

This study was part of a controlled feeding study assessing the accuracy, cost-effectiveness and acceptability of three technology-assisted dietary assessment methods. The details of the study protocol and main outcomes have been published previously^(29,30)^. A convenience sample with approximately equal numbers of men and women were recruited from Curtin University staff and students in Perth, Australia, by email advertisement. Exclusion criteria were serious illnesses or medical conditions, pregnancy, special dietary requirements or dietary restrictions due to food allergies, intolerances or dieting to lose weight.

### Study design

The study used a cross-over design, and all participants were asked to attend 3 feeding days 1 week apart and subsequently on the following day complete each of three technology-assisted dietary assessment methods to report 1 d of dietary intake: (1) Automated Self-Administered Dietary Assessment Tool (ASA24)^(31)^; (2) Intake24^(32)^; and (3) Interviewer-Administered Image-Assisted 24-h dietary recall (IA-24HR)^(29)^. Participants were randomised for the order in which they were asked to complete the three methods. The target sample size was 150 participants to allow for a 20 % dropout, while maintaining 90 % power at a 5 % significance level when the true difference between any two mean differences between estimated and true energy intake was zero.

### Procedures

Prior to the first feeding day, participants were asked to complete an online demographic questionnaire, including questions on age, sex and highest level of educational attainment. Participants also completed computerised versions of cognitive tasks online. Based on the literature and researcher judgement, we selected a range of cognitive measures considered to align with neurocognitive processes involved in 24HR, covering working memory, vividness of visual imagery, cognitive flexibility and visual attention. Participants completed the tasks described below in the following order: the Trail Making Test, the Wisconsin Card Sorting Test, the Visual Digit Span and the Vividness of Visual Imagery questionnaire.

#### Trail Making Test

The Trail Making Test^(33)^ assesses visual attention and complex visual scanning^(12)^ and overall fluid cognitive ability^(34)^. Participants were asked to draw lines in specific, predetermined sequences from nodes to nodes on a screen, as quickly and as accurately as possible. If a participant made a line to an incorrect target, they were informed of the error and directed to return to the last correct target and try again. The test included four trials and took approximately 5 min to complete. The outcome measure used was the time spent on the task, which reflects a combination of speed and accuracy.

#### Wisconsin Card Sorting Test

The modified Wisconsin Card Sorting Test^(35)^ assesses cognitive flexibility. Cognitive flexibility, one of the brain’s core executive functions^(19)^, is the ability to switch thinking and behaviours in response to changing demands. It is considered to involve several cognitive processes simultaneously, namely, working memory, switching, inhibition and salience detection and attention^(36)^. On a screen, participants were asked to sort cards into four different ‘categories’. No instructions are given about the categorisation rules. Participants were informed whether each selection was correct or incorrect. The cards to sort into these piles had similar designs and varied in colour (four variants: red, green, yellow, blue), shape (four variants: triangle, star, cross, circle) and number of shapes (four variants: 1, 2, 3, 4). Categorisation rules changed mid-task without warning. Participants were required to deduce that the rule had changed and determine what the new rule was. How long participants persevere with an old rule once it no longer applies is thought to indicate the level of cognitive flexibility^(37)^. Thus, the outcome measure used was the number of accurate trials as a percentage of the total number of trials. The task takes approximately 2 min to complete.

#### Visual Digit Span (forwards/backwards)

The Visual Digit Span procedure^(38)^ measures working memory, which is the ability to hold information in your mind and manipulate it^(19)^. In this task, participants saw sequences of digits on a computer or mobile phone screen and were asked to recall them by selecting the recalled digits from a circle of digits with their mouse/finger. In the first part of the procedure, participants were asked to recall digits in a forward manner, as presented to them. In the second half of the procedure, participants were asked to recall the digits in a backward manner. Depending on performance, participants moved up a level or down a level to longer or shorter spans. The assessment ended after fourteen trials and was expected to take approximately 10–15 min. The outcome measure used in the analyses was the last digit span a participant got correct before making two consecutive errors, and as recommended by Reynolds (1997), forward and backward recall measures were considered separately^(39)^.

#### Vividness of Visual Imagery questionnaire

The Vividness of Visual Imagery questionnaire measures the strength of visual imagery, which indicates how well a participant can conceptualise visual memory. Participants were asked to complete a sixteen-item self-administered questionnaire twice: first with eyes open, then with eyes closed immediately before answering each question. Participants were asked to imagine people/scenes and rate the vividness of these mental images using a 5-point scale with a glider (1 = perfectly clear and vivid as if I was actually seeing it, 2 = reasonably clear and vivid, 3 = moderately clear and vivid, 4 = vague and dim, 5 = no image at all)^(40)^. The questionnaire takes approximately 10–15 min to complete. Possible scores range from 32 to 160, with lower scores indicating stronger visual imagery. The outcome measure used was the total score.

### Dietary intake measurement error

#### True intake

Participants attended the food laboratory for breakfast, lunch and dinner on 3 separate days, 1 week apart, selecting items from a menu, subsequently consuming meals *ad libitum*, then leaving the laboratory between meals. All food and beverage items were inconspicuously weighed before serving to participants and, after tray return, to calculate the amount of each food consumed. The weight of each food consumed was entered into nutrition analysis software (FoodWorks 10, Xyris Software) linked to the AUSNUT 2011–13 food nutrient database^(41)^ to estimate energy intake. Error in energy estimation (rather than another component such as protein) was chosen to best reflect total dietary intake and to be comparable with other studies on dietary intake measurement error.

#### Reported intake

Each day subsequent to the feeding day, participants completed a 24HR interview, each time via a different technology-assisted dietary assessment method (ASA24^(31)^; Intake24^(32)^; IA-24HR). A 24HR is designed to capture detailed information on foods and beverages consumed in the previous day or previous 24 h. Details of each of these methods have been described elsewhere^(29)^. Briefly, all methods were based on the multiple-pass method, a structured interview format with specific probes to enhance recall of food details and amounts. ASA24 and Intake24 were self-administered, while IA-24HR^(42–45)^ was interviewer-administered and included interviewers and researchers viewing images participants had captured of the foods and beverages they had consumed. With ASA24 and Intake24, portion size estimation was completed by participants, while in IA-24HR, participants’ estimations of portion sizes were verified by interviewers. Food and beverages reported using ASA24 and Intake24 were automatically linked to food codes and gram weights from the AUSNUT 2011–13 food nutrient database^(41)^ for the estimation of energy intake. With IA-24HR, two coders individually entered all IA-24HR recalls into nutrition analysis software (FoodWorks 10, Xyris Software), and after data entry corrections, the average energy intake of the two datasets was used in the analysis.

### Data analyses

Any items reported at eating occasions outside of the food laboratory were identified by manually examining eating times and eating occasions and were excluded from the data before analysis. For each participant, the difference between true and reported total daily energy intake was calculated for each 24HR method. Then the percentage error between reported and true energy intakes was calculated as (reported – true)/true × 100, and absolute values were used as the outcome variable in analyses. Spearman’s rank correlation was used to assess the association between task scores, and *t* tests were used to assess the association between task scores and demographic characteristics (age, sex and educational attainment). The interaction between tasks and demographic characteristics was assessed for associations with percentage error in estimated energy intake using the Lasso regression procedure in STATA 18 (StataCorp). Univariate linear regression was used to assess the association of each cognitive task outcome measure with percentage error in energy intake. In the multivariate model, stepwise regression was conducted with a cut-off of 0·1 on the likelihood ratio test. This cut-off was used to prevent the loss of potentially important variables, which may have occurred should a more stringent cut-off be used. Age, sex and educational attainment were retained in all models based on previous associations with task scores^(12,46)^, while BMI was retained based on associations with error in estimation of energy intake^(21)^. The total number of food and beverage items consumed was also included as a covariate since greater variety within meals has resulted in more difficulty for participants in remembering their diet as compared with those with more regular eating routines^(47)^. Recall completion time and method order (ASA24, Intake24 and IA-24HR) were also included as covariates in all models. A sample size of 139 participants produces a two-sided 95 % CI of +/– 8 % in the percentage error in energy intake. Statistical analyses were conducted in IBM SPSS Statistics 28 (IBM Corp) and STATA 18 (StataCorp). The STROBE-nut checklist (online Supplementary Table 1) guided the reporting of this study^(48)^.

## Results

### Participant characteristics

**Figure. 1.**
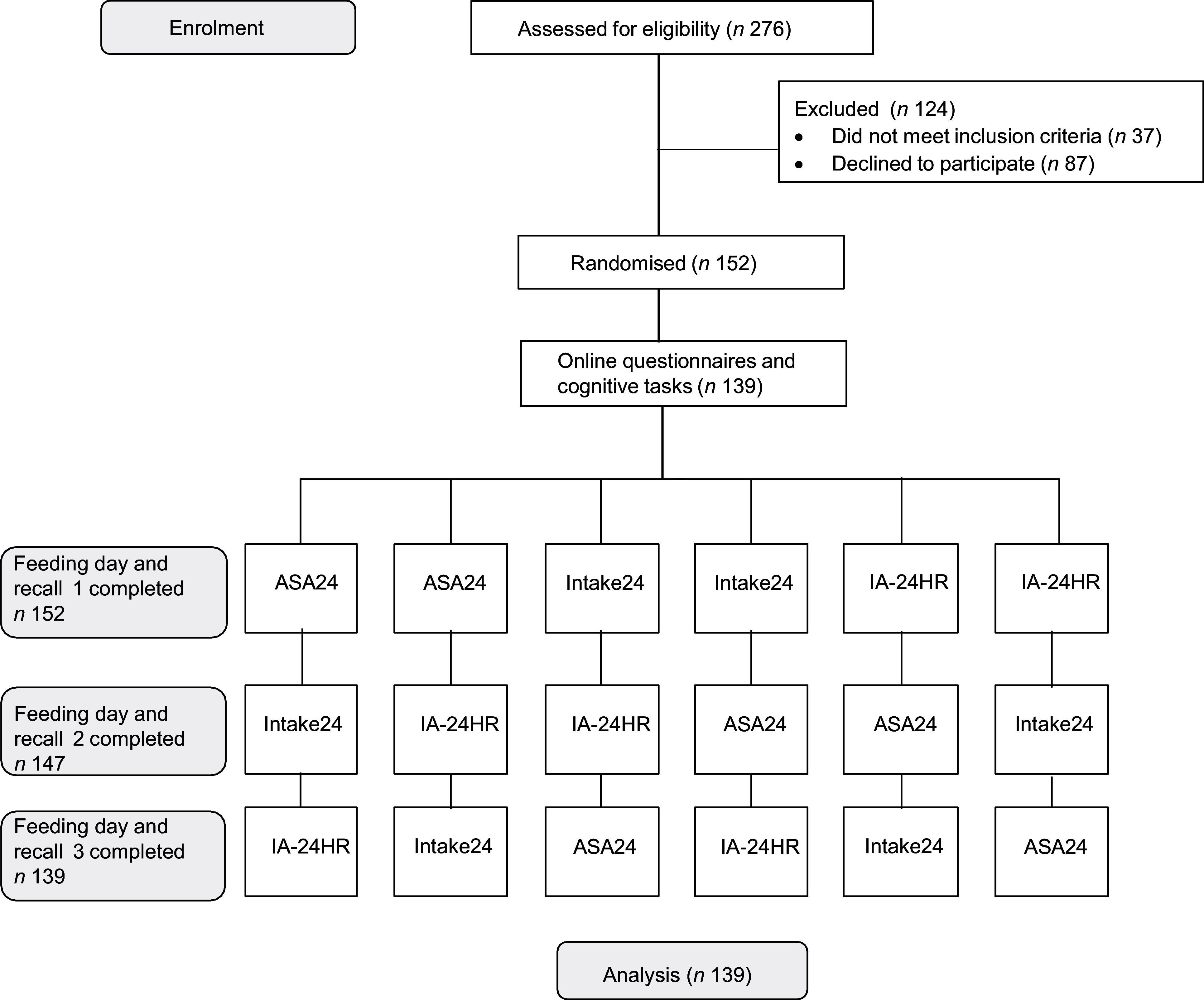
Study flow chart on enrolment, randomisation and study design. ASA24, feeding day followed by completion of Automated Self-Administered Dietary Assessment Tool (ASA24®)-Australia; Intake24, feeding day followed by completion of Intake24-Australia; IA-24HR, feeding day including capture of images of meals using mobile Food Record app, followed by completion of Interviewer-Administered Image-Assisted 24-h dietary recall.

A total of 152 participants enrolled in the study, and 139 participants completed at least one cognitive task (Fig. 1). Of these, 138 completed the Card Sorting Test, 138 completed the Trail Making Test, 129 completed the Digit Span and 126 completed the Vividness of Visual Imagery questionnaire. Participants’ ages ranged from 19 to 65 years, with a mean of 32·3 years (sd 10·6) (Table 1). A greater proportion of participants identified as ethnically Asian (54·7 %) relative to the general population^(49)^. Most participants held a bachelor’s degree or higher (72·7 %).

**Table 1.** Characteristics of participants in ACE-TADA, who completed at least one cognitive task, *n* 139 (Mean values and standard deviations; numbers and percentages; median values and interquartile ranges)

Characteristic	Summary statistics	
	Mean	sd
Age (years), mean (sd)	32·3	10·6
BMI (kg/m^2^), mean (sd)	25·7	5·4
	*n*	%
Sex, *n* (%)		
Man	80	57·6 %
Woman	54	38·8 %
Other or prefer not to say	5	3·6 %
Ethnicity, *n* (%)		
White	57	41·0 %
Aboriginal or Torres Strait Islander	1	0·7 %
Asian	76	54·7 %
Other	5	3·6 %
Annual household income ($ AUD), *n* (%)		
Less than $60 000	37	26·6 %
$60 000–$149 999	57	41·0 %
$150 000 or above	29	20·9 %
Don’t know or prefer not to answer	16	11·5 %
Highest level of education attained, *n* (%)		
School or diploma	38	27·3 %
University bachelor’s degree or higher	101	72·7 %
	Median	IQR
Task scores, median (IQR)		
Perseverative accuracy (%)*	58·3	45·8–70·8
Maximum forward Visual Digit Span^†^	7·0	6·0–8·0
Maximum backward Visual Digit Span^‡^	7·0	5·0–8·0
Time to complete Trail Making Test (seconds)	115·6	90·0–148·0
Vividness of visual imagery (total score)^§^	74·5	57·0–93·0
Absolute error (%) in energy intake estimation, median (IQR)		
ASA24	17·8	7·7–29·6
Intake24	14·6	8·8–26·4
IA-24HR	15·4	7·2–27·6

ASA24, Automated Self-Administered Dietary Assessment Tool; IA-24HR, Interviewer-Administered Image-Assisted 24-h dietary recall.

* Number of accurate trials as a percentage of the total trials in the card sorting task.

† Maximum forward digit span achieved (the last digit span a participant got correct before making two consecutive errors).

‡ Maximum backward digit span achieved (the last digit span a participant got correct before making two consecutive errors).

§ Possible scores range from 32 to 160, with lower scores indicating stronger visual imagery.

### Associations between task scores

Greater time spent in the Trail Making Test was associated with lower accuracy on the Card Sorting Test (Rho = –0·236, *P* = 0·005) (Table 2). Greater time spent to complete the Trail Making Test was associated with less vivid visual imagery (Rho = 0·239, *P* = 0·007). Maximum forward and backward Visual Digit Spans were associated with one another (*P* < 0·001). No other associations were observed between task scores.

**Table 2.** Correlation matrix of associations between task scores

Cognitive task score		Perseverative accuracy (%)*	Maximum forward digit span^†^	Maximum backward digit span^‡^	Time to complete trail-making test (seconds)	Vividness of visual imagery (total score)^§^
Perseverative accuracy (%)*	Rho	1				
	*P*	.				
Maximum forward digit span^†^	Rho	–0·075	1			
	*P*	0·40	.			
Maximum backward digit span^‡^	Rho	–0·151	0·368	1		
	*P*	0·09	< 0·001	.		
Time to complete trail-making test (seconds)	Rho	–0·236	–0·086	0·062	1	
	*P*	0·005	0·33	0·489	.	
Vividness of visual imagery (total score)^§^	Rho	–0·024	–0·102	0·114	0·239	1
	*P*	0·79	0·26	0·205	0·007	.

* Number of accurate trials as a percentage of the total trials in the card sorting task.

† Maximum forward digit span achieved (the last digit span a participant got correct before making two consecutive errors).

‡ Maximum backward digit span achieved (the last digit span a participant got correct before making two consecutive errors).

§ Possible scores range from 32 to 160, with lower scores indicating stronger visual imagery.

### Associations between test scores and demographics

Men spent more time than other sexes completing the Trail Making Test (+18·0 s, 95 % CI 2·3, 34·0; *P* = 0·025), as did participants older than the mean age (+17·8 s, 95 % CI 2·3, 33·3; *P* = 0·025). However, older participants recalled longer backward Digit Spans than younger participants (+1·2, 95 % CI 0·1, 2·2; *P* = 0·033). Participants with university education (*v*. those without) obtained lower perseverative accuracy scores in the card sorting task (–6·0 percentage points, 95 % CI 0·5, 12·2; *P* = 0·035).

### Associations of task scores and percentage error in energy intake estimation

Using ASA24, the amount of time spent completing the Trail Making Test (seconds) was positively associated with percentage error in energy intake estimation (B 0·13, 95 % CI 0·04, 0·21) (Table 3). This equates to an additional 7·6 % (95 % CI 2·6, 12·6) error for every extra minute spent on the Trail Making Test. Maximum backward Digit Span was retained in the final multivariate model for ASA24 because of its contribution to *R* ^2^ (0·026) but was not statistically significant at the 5 % level (B −1·16, 95 % CI −2·44, 0·13, *P* = 0·077). Cumulatively, time spent completing the Trail Making Test, the maximum backward Digit Span and covariates accounted for 13·6 % of the variation present in energy intake estimation error.

**Table 3.** Associations between cognitive task scores and percentage error in estimated energy intake, *n* 139

	ASA24				Intake24				IA-24HR			
Independent variable*	B	se	*P*	*R* ^2^ change	B	se	*P*	*R* ^2^ change	B	se	*P*	*R* ^2^ change
Univariable model												
Perseverative accuracy (%)^†^	0·08	0·1	0·45	0·4 %	–0·04	0·11	0·68	0·1 %	–0·05	0·09	0·59	0·2 %
Maximum forward Visual Digit Span^‡^	–0·17	0·73	0·82	0·0 %	0·29	0·77	0·71	0·1 %	–0·51	0·62	0·40	0·6 %
Maximum backward Visual Digit Span^§^	–0·41	0·56	0·47	0·4 %	0·04	0·6	0·95	0·0 %	–0·13	0·48	0·80	0·1 %
Time to complete Trail Making Test (sec)	0·10	0·03	0·003	6·5 %	0·06	0·04	0·09	2·1 %	0·00	0·03	0·87	0·0 %
Vividness of visual imagery (total score)^||^	0·03	0·06	0·62	0·2 %	0·03	0·06	0·67	0·1 %	–0·05	0·05	0·31	0·9 %
Univariable model, adjusted^¶^												
Perseverative accuracy (%)^†^	0·06	0·11	0·62	3·6 %	–0·06	0·11	0·60	10·7 %	–0·01	0·09	0·93	8·9 %
Maximum forward Visual Digit Span^‡^	–0·37	0·79	0·64	4·4 %	0·02	0·8	0·98	11·5 %	–0·85	0·64	0·19	12·3 %
Maximum backward Visual Digit Span^§^	–0·98	0·66	0·14	6·1 %	0·26	0·66	0·69	11·6 %	0·00	0·54	1·00	10·9 %
Time to complete Trail Making Test (sec)	0·12	0·04	0·002	11·0 %	0·08	0·04	0·05	13·1 %	0·02	0·03	0·56	9·6 %
Vividness of visual imagery (total score)^||^	0·01	0·07	0·87	4·3 %	0·04	0·07	0·59	12·1 %	–0·04	0·05	0·46	14·0 %
Multivariable model^¶^												
Perseverative accuracy (%)^†^												
Maximum forward Visual Digit Span^‡^												
Maximum backward Visual Digit Span^§^	–1·16	0·65	0·08	2·6 %								
Time to complete Trail Making Test (sec)	0·13	0·04	0·003	6·6 %	0·10	0·04	0·02	4·2 %				
Vividness of visual imagery (total score)^||^												
Total *R* ^2^				13·6 %				15·8 %				

ASA24, Automated Self-Administered Dietary Assessment Tool; IA-24HR, Interviewer-Administered Image-Assisted 24-h dietary recall.

* Dependent variable is the absolute error in energy intake as a percentage of true intake.

† Number of accurate trials as a percentage of the total trials in the card sorting task.

‡ Maximum forward digit span achieved (the last digit span a participant got correct before making two consecutive errors).

§ Maximum backward digit span achieved (the last digit span a participant got correct before making two consecutive errors).

|| Possible scores range from 32 to 160, with lower scores indicating stronger visual imagery.

¶ Adjusted for the covariates: age (continuous); male sex (*v*. not); BMI (continuous); university educated (*v*. not); total number of food/beverage item reported (continuous); order of administration of recall methods; time taken to complete recall (continuous).

The amount of time spent completing the Trail Making Test (seconds) was also positively associated with percentage error in energy intake estimation (B 0·10, 95 % CI 0·02, 0·19) using Intake24 (Table 3). This equates to an additional 6·2 % (95 % CI 1·0, 11·4) error for every extra minute spent on the Trail Making Test. No other cognitive task scores were present in the final multivariate model for Intake24, which accounted for 15·8 % of the variation present in the error in energy intake estimation.

With IA-24HR, no cognitive task scores were associated with error in energy intake estimation, either in univariate or multivariate models. No significant interaction terms were identified between tasks or between tasks and demographic characteristics.

## Discussion

In this study, individual variation in neurocognitive processes, specifically the time spent on a task measuring visual attention, was associated with dietary intake estimation error using two self-administered web-based 24HR. In contrast, the error in dietary intake estimated using IA-24HR, where participants viewed images of their foods and beverages during the recall process, was not associated with any cognitive task scores. This exploration of cognitive tasks and measurement error in the context of dietary assessment is highly novel, with significant potential to impact on practice. Researchers have cited cognitive load as a barrier in the collection of accurate dietary intake data^(9–11)^, and this study provides empirical evidence for this assertion.

Longer time spent completing the Trail Making Test was associated with greater error in energy intake estimation using ASA24 and Intake24. Completion of the Trail Making Test requires several neurocognitive processes, including focusing attention, sequencing and attentional shifting^(34)^. These processes contribute to the skills of planning and reasoning, problem-solving and organisation, which are part of executive function^(50)^. Completion of the Trail Making Test also requires nonexecutive components such as visual spatial ability and processing speed^(50)^. It is conceivable that each of these neurocognitive processes and skills is involved in a 24HR, but it is unclear whether one, multiple or all are underlying the association with energy estimation error; thus, further research is recommended.

Absolute error in energy intake estimation was similar across the methods; however, a detailed study we published elsewhere illustrated distinct patterns in measurement error for each of the methods^(30)^. The final regression models explained 13·6 % (ASA24) and 15·8 % (Intake24) of the variance in energy estimation error. Although these values are similar, the contribution of covariates differed between ASA24 and Intake24. With Intake24, 4·0 % of the variation in error was explained by the Trail Making Test, while more than 10 % was explained by covariates. With ASA24, however, the Trail Making Test and backward Digit Span contributed to 9·2 % of the variance in energy estimation error. The contribution of cognition to estimation error is a greater proportion of the variance explained than is typically observed in studies investigating the impact of other individual characteristics. For example, in the US population, weight status accounted for 5 % (men) and 7 % (women) of the variance in estimated energy intake from a 24HR relative to BMR^(51)^. In another US study, psychosocial and dietary factors accounted for 2 % of variance in error in a combined estimate from a 24HR, food record and FFQ^(52)^. From the perspective of developing inclusive, effective and accurate dietary assessment instruments, ideally, the variation in error explained by cognitive task scores would be minimal. However, this study suggests that with both ASA24 and Intake24, individual variation in neurocognitive processes may be as important and currently overlooked factor in measurement error as BMI or sex. Thus, it is recommended that large-scale surveillance of population dietary intakes using 24HR methods includes a measure of cognitive skills in order to identify any sub-groups in which data may be less reliable.

With ASA24, the maximum backward digit span recalled was retained in the final multivariate model because of its contribution to *R* ^2^, although it was not statistically significant (*P* = 0·08). Despite similarities in ASA24 and Intake24, maximum backward digit span did not appear in the final multivariate model for Intake24. Both forward and backward digit span tasks assess working memory capacity. However, the backward digit span is thought to invoke neurocognitive processes not involved in the recall of forward digit spans^(39)^, such as visuospatial imaging processes and the manipulation and transformation of the visual and spatial information held in the working memory^(53)^. Conceivably, such neurocognitive processes are invoked during a self-administered 24HR, particularly during portion size estimation using standardised images. For example, when a participant is asked to report a portion size using a standardised image of a food that is different from the food they consumed and served on a plate that is different from the size of their own plate, there will be a need to manipulate and transform information in their working memory. Refinement of 24HR instruments should aim to minimise the need for visuospatial image processing and manipulation since individual variation in these processes may contribute to the systematic measurement error associated with portion size estimation.

No cognitive task scores were included in the final regression model for IA-24HR. This may be explained by the processes involved in the administration of the interviewer-administered IA-24HR, which was distinct from the self-administered 24HR ASA24 and Intake24. Images collected by participants showing their food and beverages were viewed during the IA-24HR. In addition, the combined judgements of interviewers and participants when identifying food descriptions and portion sizes may have reduced the cognitive burden on participants. Supplementing self-administered web-based 24HR such as ASA24 and Intake24 with images collected by participants may be an effective strategy in reducing cognitive burden and therefore reducing the contribution of individual variation in neurocognitive processes to systematic measurement error. To test this theory, future studies exploring the effect of supplementing self-administered 24HR with participant-captured images could also include some simple cognitive tasks and compare associations with error in the presence and absence of images.

There are several strengths and limitations in relation to this study. The cross-over methodology used in this study allowed direct comparison of 24HR methods in the same individuals. Although, in theory, a learning effect may have been present as participants completed each of the 24HR methods, no associations with method order and energy estimation error were observed. This study design was therefore ideal to compare 24HR methods and correlates of energy estimation error. The paucity of evidence on the role of neurocognitive processes in the measurement error inherent in self-reported dietary intake is a gap that warrants attention. This study is among the first to explore the association of individual variation in the neurocognitive processes of adults with energy estimation error. A vast body of literature exists on the associations of demographic factors such as sex and age with error^(8,21,54)^, but these factors are likely crude proxies for the underlying processes that truly drive variation in error in energy estimation from self-reported dietary intake. Understandably, the complex neurocognitive processes involved in the recall of dietary intake are difficult to measure reliably and accurately, and this may explain why they are rarely explored in the dietary assessment literature. It is hoped that this study will inspire the further exploration of this area, leading to the development and refinement of 24HR.

It is likely that the cognitive burden associated with recalling intake will vary according to the type, complexity and physical form of the food consumed. For example, it is conceivable that the more components present in a meal, the more difficult it is to recall. In the present study, we attempted to account for meal complexity by including the number of items reported in each 24HR as a covariate in the regression model. However, we did not account for the variation in the physical form of the food and recommend that this under-researched area^(55)^ is investigated in future research using food and beverage intake data from controlled feeding studies.

Test–retest reliability studies of complex executive tasks indicate that scores are not perfectly reproducible^(56)^. For example, in an evaluation of test–retest reliability of the visual imagery scale based on seven studies with an interval of 3–7 weeks, the mean reliability coefficient was 0·74^(57)^. One possible explanation is that executive control processes are strongest when the task is novel^(56)^. Another explanation is that stress impacts memory retrieval^(58,59)^ and influences attentional processes, contributing to memory distortions^(60)^. The order of administration of cognitive tasks in this study was not randomised, in that all participants completed the tasks in the same order (Trail Making Test, Wisconsin Card Sorting Test, Visual Digit Span and Vividness of Visual Imagery questionnaire). This may have resulted in fatigue towards the end of the batch of tasks, with better quality data obtained from tasks administered first.

Finally, some limitations were present related to the study population. We did not screen for cognitive impairment among participants, so it is possible this was present to some extent in our sample. Cognitive impairment is associated with ageing and is more prevalent among adults aged 65 years and above as compared with younger adults^(61)^. Therefore, it is unlikely to have had a major impact on our findings. The place of consumption was standardised for all participants (the food laboratory). However, the 24HR typically took place at the workplace or at home. Data on the place where the 24HR was conducted was not collected systematically; thus, any variation in recall associated with the participants’ environment was not accounted for in the analysis. Furthermore, our self-selecting sample of university staff and students was not representative of the general population. It is unclear exactly how this may have affected the results and interpretation in the present study. It is possible that among our relatively homogenous study population, variation in both neurocognitive process and variation in error was lower than would be observed in the general population, thus attenuating the effect size of our findings. Replication of the findings of this study in other study populations is needed to help confirm the impact of variation in neurocognitive processes on energy estimation error in 24HR and to elucidate whether the Trail Making Test or other tasks or tests best align with and measure the processes involved in a 24HR.

Several recommendations can be made as a result of this research. More focus on the underlying mechanisms driving dietary assessment measurement error, such as neurocognitive skills, will enable the development and refinement of effective and inclusive 24HR instruments. Therefore, researchers developing, refining and using 24HR instruments should consider administering cognitive tasks as in this study to understand whether variation in cognitive processes is associated with error in their instrument and strive to reduce this association when present. Reducing the association between cognitive skills and measurement error may require a move away from a 24HR, which relies heavily on participant memory, but more research is needed to confirm this. For example, the use of prospective dietary assessment instruments or image-assisted 24HR instruments is a possible solution^(30)^.

### Conclusion

This cross-over evaluation of three technology-assisted 24HR instruments, using a controlled feeding study design, assessed the association of individual variation in neurocognitive processes with error in 24HR energy estimation. The time spent on a trail-making task measuring visual attention, sequencing and attentional shifting was associated with energy estimation error in the self-administered web-based 24HR ASA24 and Intake24. In contrast, energy estimation error using IA-24HR, which used images collected by participants during the recall process, was not associated with any cognitive task scores. Further investigation into the impact of neurocognitive processes in relation to 24HR energy estimation error is required to inform the further development of self-administered 24HR instruments with reduced systematic measurement error. Future population nutrition surveillance using 24HR should consider the inclusion of cognitive assessments to indicate data reliability.

## Supporting information

Whitton et al. supplementary material Whitton et al. supplementary material

## Supplementary material

For supplementary material accompanying this paper visit https://doi.org/10.1017/S000711452500042X
